# The Spike of SARS-CoV-2: Uniqueness and Applications

**DOI:** 10.3389/fimmu.2021.663912

**Published:** 2021-07-08

**Authors:** Ranjith Kumavath, Debmalya Barh, Bruno Silva Andrade, Madangchanok Imchen, Flavia Figueira Aburjaile, Athira Chakkadath, Diego Lucas Neres Rodrigues, Sandeep Tiwari, Khalid J. Alzahrani, Aristóteles Góes-Neto, Marianna E. Weener, Preetam Ghosh, Vasco Azevedo

**Affiliations:** ^1^ Department of Genomic Science, School of Biological Sciences, Central University of Kerala, Kasaragod, India; ^2^ Centre for Genomics and Applied Gene Technology, Institute of Integrative Omics and Applied Biotechnology (IIOAB), Nonakuri, Purba Medinipur, West Bengal, India; ^3^ Laboratório de Genética Celular e Molecular, Departamento de Genetica, Ecologia e Evolucao, Instituto de Ciências Biológicas, Universidade Federal de Minas Gerais, Belo Horizonte, Brazil; ^4^ Laboratório de Bioinformática e Química Computacional, Departamento de Ciências Biológicas, Universidade Estadual do Sudoeste da Bahia (UESB), Jequié, Brazil; ^5^ Department of Clinical Laboratories Sciences, College of Applied Medical Sciences, Taif University, Taif, Saudi Arabia; ^6^ Laboratório de Biologia Molecular e Computacional de Fungos, Departamento de Microbiologia, Instituto de Ciências Biológicas, Universidade Federal de Minas Gerais (UFMG), Belo Horizonte, Minas Gerais, Brazil; ^7^ Clinical Research Center, Oftalmic, CRO, Moscow, Russia; ^8^ Department of Computer Science, Virginia Commonwealth University, Richmond, VA, United States

**Keywords:** SARS-CoV-2, COVID-19, spike protein, mutations, diagnostics, drugs, vaccines

## Abstract

The Spike (S) protein of the SARS-CoV-2 virus is critical for its ability to attach and fuse into the host cells, leading to infection, and transmission. In this review, we have initially performed a meta-analysis of keywords associated with the S protein to frame the outline of important research findings and directions related to it. Based on this outline, we have reviewed the structure, uniqueness, and origin of the S protein of SARS-CoV-2. Furthermore, the interactions of the Spike protein with host and its implications in COVID-19 pathogenesis, as well as drug and vaccine development, are discussed. We have also summarized the recent advances in detection methods using S protein-based RT-PCR, ELISA, point‐of‐care lateral flow immunoassay, and graphene-based field-effect transistor (FET) biosensors. Finally, we have also discussed the emerging Spike mutants and the efficacy of the Spike-based vaccines against those strains. Overall, we have covered most of the recent advances on the SARS-CoV-2 Spike protein and its possible implications in countering this virus.

## Introduction

The coronaviruses belong to the Nidovirales order, Coronaviridae family, which is divided into Alphacoronavirus, Betacoronavirus, Deltacoronavirus, and Gammacoronavirus genera (1). To date, the Betacoronavirus is the most studied genera, as it includes airborne viral species, such as severe acute respiratory syndrome–related coronaviruses (SARS-CoV) and Middle East respiratory syndrome coronaviruses (MERS-CoV), which can infect humans. In 2019, a new coronavirus, named as severe acute respiratory syndrome coronavirus (SARS-CoV-2), emerged in Wuhan, Hubei province, China (2, 3). Despite the high similarity (88%) of the viral genome with SARS-like viruses (4), the new species exhibit an increased transmission rate in human populations (5). In fact, the World Health Organization (WHO) has declared the syndrome associated with SARS-CoV-2 as a public health emergency due to its rapid global spread and declared as COVID-19 pandemic. Since then, several research groups have focused on characterizing the molecular mechanisms involved in the infection process to identify therapeutic and prophylactic targets (6). Among the main structural proteins encoded by the SARS-CoV-2 genome, the Spike (S) protein is considered fundamental in the pathogenesis, transmission, and virulence of the virus because it binds to the human host cell membrane by interacting with the host angiotensin-converting enzyme 2 (*h*ACE2) receptor (7, 8).

Given the importance of the S protein in the context of the COVID-19 pandemic, the objective of this study is to conduct an in-depth review based on available literature on the novel coronavirus (2019-SARS-CoV-2) S protein. Additionally, we applied two different meta-analysis approaches based on keywords associated with the S protein to generate the outline of this article and introduce the covered topics.

## Methods and Results of Meta-Analysis

For the meta-analysis, we selected all the publications available in the PubMed database that matched the search for keywords (chloroquine, diagnostic, nCoV, Spike, treatment, vaccine), and a file containing the publication dates was obtained for each keyword. Subsequently, a filter was applied to obtain the number of publications per month, which made it possible to analyze the average number of publications per year, before and during the pandemic period characterized from January 01, 2020, until April 20, 2021. Thus, we estimated the statistical difference before the pandemic period and during the ongoing SARS-CoV-2 outbreak. The quantitative analysis of the number of publications in each period has little chance of bias, as it considers the entire set of data available in the database. Because the goal of our study was not to observe any specific clinical event, exclusion criteria were not considered; therefore, all the studies that were related to the coronavirus were added. This makes the meta-analysis presented here representative to perform comparative analyses. To carry out the statistical analysis, the effect sizes were performed by Hedges test. Thus, the values of the mean and standard deviation of publications were initially calculated. After obtaining the values of the effect sizes, the summary of the results and the final plot were generated in R using the publicly available “meta” package. The random effects model was selected because of the large differences found when comparing the before and during pandemic periods.

**Figure 1:**
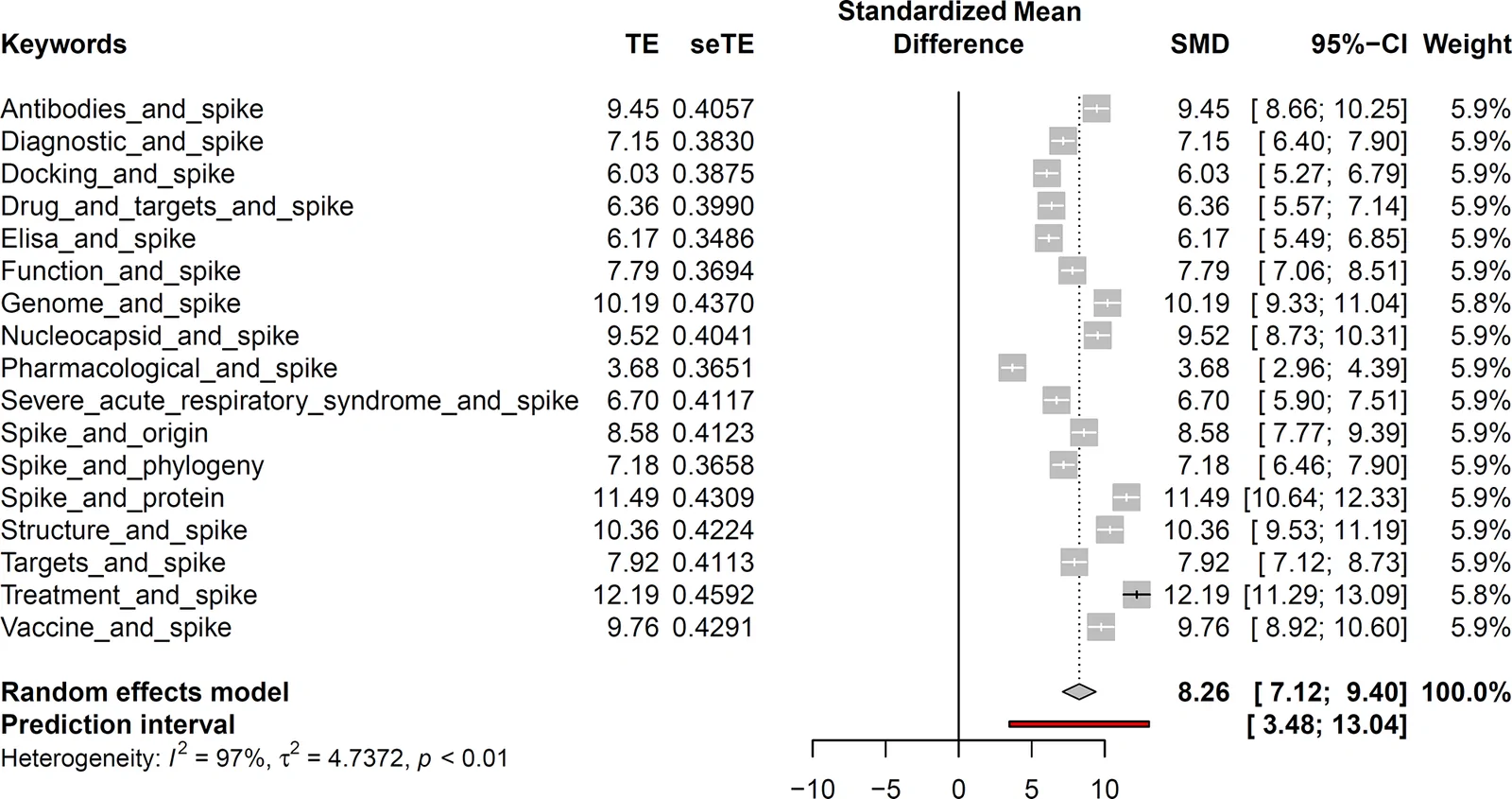
Brief meta-analysis study based on the six keywords used. The white lines represent a large dataset for the selected keywords.

The values found in the different periods considered in our study are displayed in **Supplementary Tables S1** and **S2**. As shown in **Figure 1**, among the results from PubMed related to our six keywords (namely “Chloroquine,” “Diagnostic,” “nCoV,” “Spike,” “Treatment,” “Vaccine”), “Vaccine” and “Spike” obtained the highest values of estimated treatment effect (TE) (14.44 ± 0.44 and 11.64 ± 0.43, respectively). This showed that both of these terms would obtain accurate and reliable results. According to the scientific articles in the literature, these two terms were also the most studied (high weights 16.6%). The confidence interval (CI), represented by the diamond, showed that the analysis was very precise and summarized the results well. The CI values, representing the estimates of the effect, and the heterogeneity value showed us that the results between the studies are very divergent (*I* ^2^ = 98%).

**Figure 2:**
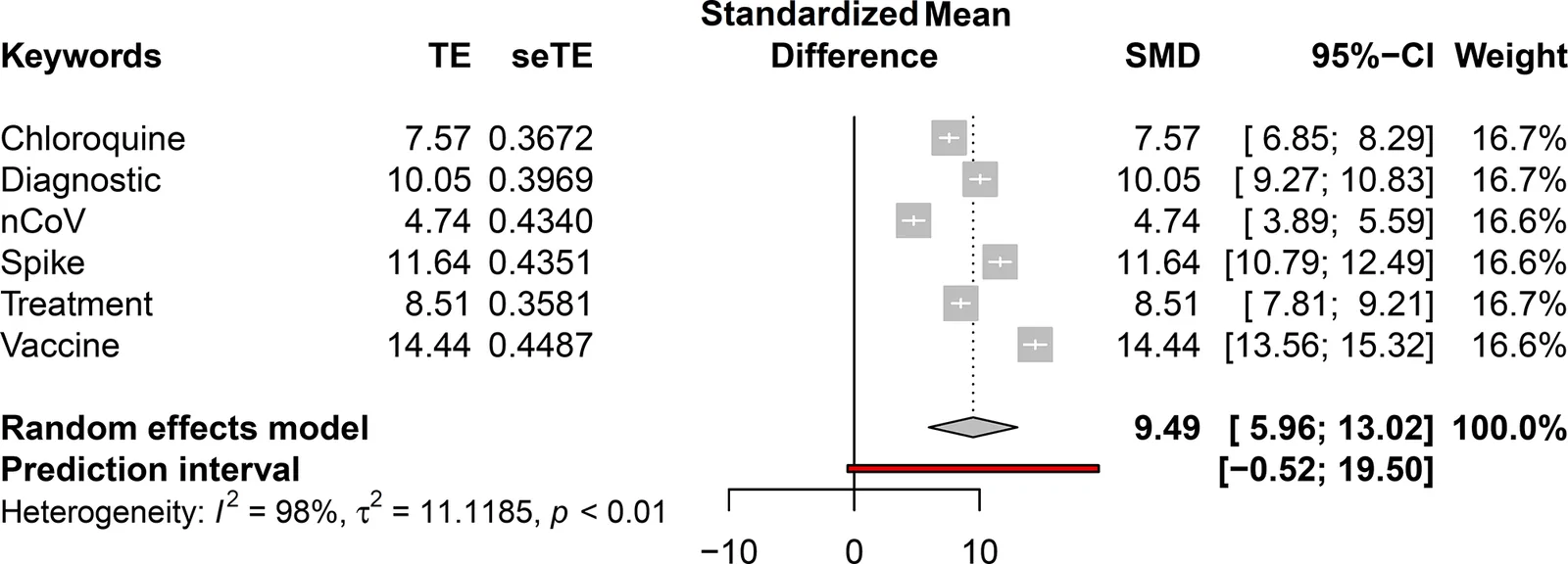
Introductory meta-analysis based on the average number of publications before and during the pandemic period.

Afterward, a second meta-analysis methodology was implemented to define the outline of this review article (**Figure 2**). For this analysis, each selected keyword-associated Spike protein publication was assembled, and then, a statistical test was performed on the monthly publications associated with the keywords in accordance with the data before (until December 31, 2019) and during the pandemic. In this case, the hypothesis was that there would be a difference between the publications associated with these terms before and during the pandemic. In order to obtain the effect size and its standard error for each sample, the Hedge’s g test was performed. Therefore, the R package *Effect Size Computation for Meta-Analysis* (esc) v0.5.1 was used based on average values, standard deviation, and sample size within each group. Finally, the data were obtained using the Meta v4.13 R package and plotted using the forest function.

The main topics addressed in this review are to the left of **Figure 2**, which indicates the paucity of studies for the relationship between Spike and the respective terms. This motivated our study and indicated that new studies are required to achieve positive correlations and associations.

## Structure and Function of Coronavirus Spike Protein

**Figure 3:**
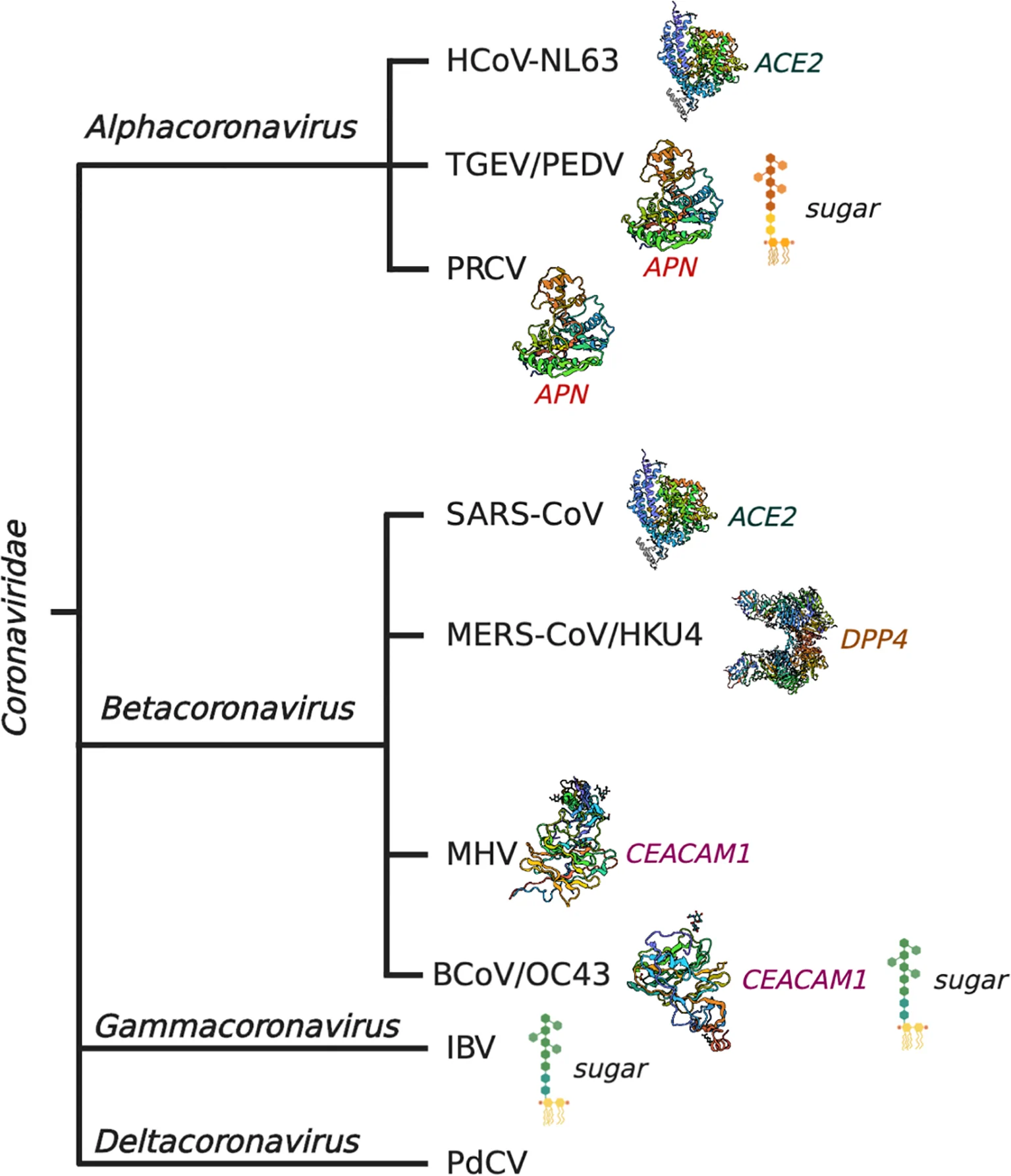
Classification of coronaviruses and their respective mechanisms of host cell adhesion and entry.

The Spike protein is a surface-anchored viral glycoprotein that comprises the second-largest open reading frame (ORF) in the Coronavirus (CoV) genome (8, 9), with approximately 200 kDa, belonging to class I viral fusion proteins (7, 10). It is the main regulator of viral attachment because of its penetration into the host cell membrane. Moreover, S protein plays a key role in defining the virulence of the virus, determining target tissues, and host diversity (11). The S protein mediates the entry of CoVs into the host cell by interacting with different receptors, depending on the CoV’s genus: *Alphacoronavirus*, *Betacoronavirus*, *Gammacoronavirus*, or *Deltacoronavirus* (**Figure 3**) (7, 12). The CoV Spike proteins from different genera can bind to the same host cell receptor. For instance, alphacoronavirus HCoV-NL63 and the betacoronavirus SARS-CoV interact with a zinc peptidase angiotensin-converting enzyme 2 (ACE2) (12, 13). On the other hand, CoVs from the same genera can also recognize different host receptors, such as MERS, which recognizes dipeptidyl peptidase 4 (DPP4) and is included in the *Betacorornavirus* genus (14–16). The CoV Spike has three main domains: a small cytoplasmic domain, an ectodomain that contains most of the elements involved in connection with the host cell, including fusion, and a transmembrane domain (7, 17). The two major domains, S1 N-terminal (S1-NTD) and S1 C-terminal (S1-CTD), are mainly involved as receptor binding domains (RBD) (7). The NTDs are responsible for sugar binding and the CTDs interact with protein receptors, such as ACE2, aminopeptidase N (APN), and DPP4 (18–20). An exception is the S1-NTD from MERS-CoV that recognizes the protein receptor carcinoembryonic antigen family (CEACAM1) (21). Furthermore, some CoV species use sugars for entry into the cell. This is the case of the transmissible gastroenteritis virus (TGEV) and porcine epidemic diarrhea virus (PEDV) under the *Alphacoronavirus* genus, bovine coronavirus (BCoV), and human coronavirus OC43 under the *Betacoronavirus* genus and infectious bronchitis virus (IBV) under *Gammacoronavirus* genus (22).

**Figure 4:**
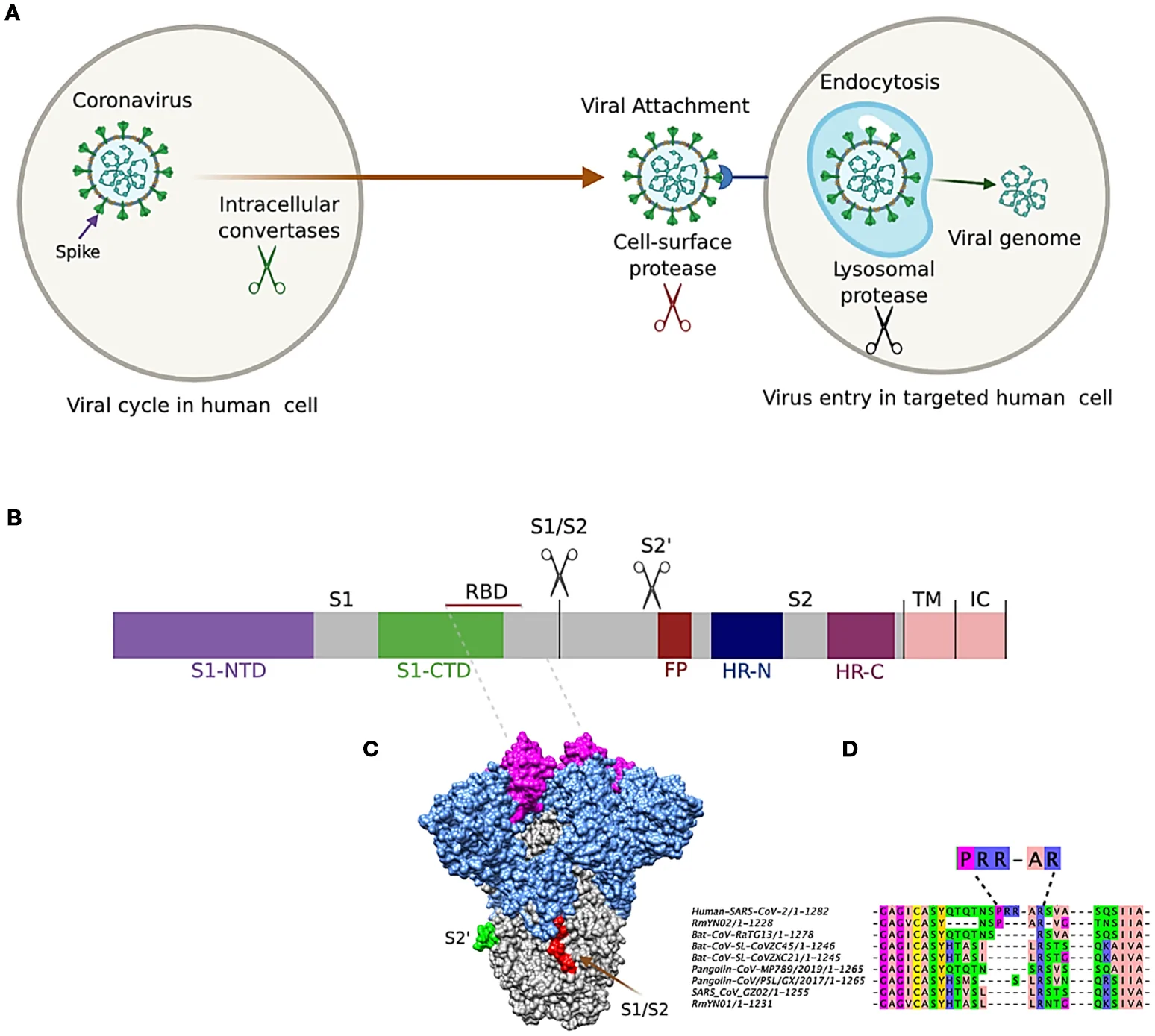
**(A)** Entry of coronavirus into host cell and the role of protease cleavage in viral replication. **(B)** Schematic description of coronavirus Spike. S1-NTD: N-terminal domain; S1-CTD: C-terminal domain; RBD: Receptor Binding Domain; S1/S2: furin/multibasic cleavage site; S2: proteolysis site; FP (fusion peptide); Heptad Repeat Regions N and C (HR-N and HR-C); Transmembrane Anchor (TM); and the Intracellular Tail (IC). S1, receptor-binding subunit; S2, membrane fusion subunit; TM, transmembrane anchor; IC, intracellular tail. **(C)** 3D representation of CoVs Spike with RBD, furin S1/S2, and S2 cleavage domains. **(D)** Sequence comparison of the SARS-CoV-2 and other SARS-CoV Spike proteins on the region at the S1/S2 boundary, indicating the RRAR motif for SARS-CoV-2.

Coronavirus Spikes are generally subject to proteolytic cleavage at specific sites by host proteases both during and after the entry into the cell, that influences both the viral membrane fusion and its life cycle (23, 24). This cleavage process is universally required in retroviruses, orthomyxoviruses, paramyxoviruses, filoviruses, and arenaviruses (25). For such cleavage, protein convertases, such as (i) Furin, can act on the cleavage site between the S1 and S2 domains (26). Moreover, trypsin can activate S protein and generates the formation of syncytium in 293/hACE2 cells (11). Other enzymes include (ii) extracellular proteases, such as elastase that acts after viral copies leave the host cells and cell surface proteases, such as type II transmembrane serine protease (TMPRSS2), and (iii) lysosomal cathepsin L and cathepsin B (**Figure 4A**).

## Structure and Function of SARS-Cov-2 Spike

In the functional state, the Spike protein is found in two distinct conformations, the pre-fusion and post-fusion conformationS (7). The SARS-CoV-2 Spike is divided into two functional units, called S1 and S2 (10, 27, 28): subunit 1 contains the receptor-binding domain (RBD), formed by 200 amino acid residues that strongly binds to *h*ACE2 (29). Within the RBD, the receptor binding motif (RBM) is responsible for direct interaction with *h*ACE2 (30) (**Figures 4B, C** **)**. Subunit 2 (S2) has two repeat regions (HR-C and HR-N), which forms a spiral over the ectodomain of the protein, separated by an inter-helical region of ~140 amino acids (28). The S1/S2 site in SARS-CoV-2 forms an exposed multibasic loop (**Figure 4C**) composed of 77 arginine residues (31). Interestingly, this region has not been found in other SARS-CoV–related coronaviruses, but they are present in the human coronaviruses OC43, HKU1, and MERS-CoV (32). Fusion of SARS-CoV-2 with lung cells is critically dependent on the S1/S2 cleavage site by furin protease, and its motif RXXR is closely related to the furin consensus sequence RX (K/R) R (32). Furthermore, the addition of an arginine residue to this motif strongly increases the syncytium formation of the host cells, indicating that the RRAR insertion in this region could enhance cell spread and pathogenicity of SARS-CoV-2 (**Figure 4D**) (32, 33).

The RBD domain of S protein is a potential target for developing vaccines and drugs, as it could block the RBD-*h*ACE2 interaction and has antigenic properties. Nonetheless, the use of S protein has difficulties. An epitope mapping using the SARS-CoV proteome shows that 70% of the peptides that elicited T-cell responses come from various structural proteins (Spike, envelope, membrane, and nucleocapsid) (34, 35). Furthermore, as compared with the SARS-CoV S protein sequence, the SARS-CoV-2 has 75% identity, but the RBD region has 73.7% identity, and the most divergent point is precisely the RBM has 50% identity (30). Interestingly, the SARS-CoV-2 RBD not only has 17.1% identity with HCoV-NL63 but also uses the receptor ACE2 (36). This lower identity makes SARS-CoV antibodies inefficient or incapable of completely neutralizing SARS-CoV-2 (30, 37, 38). SARS-CoV-2 also brings several challenges in the development of efficient antibodies or vaccines because of its dynamic mutations in RBM within the RBD (30). Therefore, the use of SARS-CoV–derived antigens with little specificity for SARS-CoV-2 may generate the “original antigenic sin” (OAS), making the immune system susceptible to emerging SARS-like viruses (37), but cannot provide SARS-CoV-2–specific immunity. However, despite the low similarities in the RBM, the similarity in the RBD may have partial protection against SARS-CoV-2 (39). Furthermore, a conserved proteolytic cleavage site in the sequence KRSFIEDLLFNKV has been found to be highly conserved in SARS-CoV-2, as well as several other coronaviruses that might be highly resistant to mutations. Therefore, it may be a strong candidate for both drug and vaccine development (40).

## Origin of SARS-Cov-2 Spike

Phylogenetic analysis of S glycoproteins has shown that SARS-CoV-2 and SARS-CoV share a similar ancestor (41). Conversely, some studies have predicted that SARS-CoV-2 Spike is highly similar to pangolins, indicating that pangolins could be the intermediate host (42–44). Furthermore, metagenomic analysis of the pangolins’ lung, spleen, lymph, and fecal samples detected SARS-CoV-2–like viral sequences only in the lung samples (73% coverage; 91% identity) (45). The authors further identified that the RBD of CoV in pangolins were different from SARS-CoV-2 in only five residues while the CoV from bat (RaTG13) displayed differences in 19 residues. The authors hypothesized that the pangolins could be an intermediate host, and the bat or the pangolins themselves could be the natural host of SARS-CoV-2. Moreover, it is also possible that the SARS-CoV-2 could have originated by the recombination of RaTG13 and pangolins CoVs (44). The emergence of novel infectious viruses could be facilitated through gene reassortment that occurs during co-infection of a host cell with multiple viruses, which facilitates crossing over. Such mutations and gene reassortment could face positive selection and lead to evolutionary arms race (46). Of the nine recombinations identified in the SARS-CoV-2 genome, six of them were identified in the gene encoding S protein (47). Despite such evidences based on S protein, nucleotide sequence identity of whole genome between SARS-CoV-2 and pangolin CoV is comparatively lower (90.23%) compared with the SARS-CoV-2 and Bat-CoV-RaTG13 (96.12%) (48). The similarity between Bat-CoV-RaTG13 and pangolin CoV was also comparatively lower (90.15%). Hence, there could be a greater possibility of SARS-CoV-2 arising from bat (48). Nonetheless, so far, there is no conclusive evidence of zoonotic origin of SARS-CoV-2 and research to find the origin of this virus needs full attention of the scientific community. The SARS-CoV-2 Spike RBD is a hotspot for adaptive mutations that enhance the binding efficiency of the virus to its human host (49), leading to the emergence of more virulent strains with greater infectivity and transmission (50).

## The Uniqueness of SARS-Cov-2 Spike

The RBD of most CoVs, including SARS-CoV-2, resides in the C terminal domain, whereas the RBD of other viruses, such as mouse hepatitis virus (MHV), is located in the N terminal domain (11). The S protein of SARS-CoV-2 also shares a mere ~76% similarity with that of SARS-CoV in the amino acid sequence (30, 51). As compared with other CoV Spike proteins, the RBD region of SARS-CoV-2 has 26% more mutations (52). The uniqueness of the SARS-CoV-2 Spike protein compared with SARS-CoV also lies in the lower (~55%) identity in the S1 domain and ~ 91% identity in the S2 domain region (53, 54). The mutations, particularly in the S protein, have significant implications in the 3D structure. The SARS-CoV-2 S protein is composed of a relatively higher number of helices (n=4) and sheets (n=10) as compared with SARS-CoV (2 helices and 5 sheets) (52, 55). Furthermore, binding of the SARS-CoV-2 to the *h*ACE2 is also more rigid with a higher number of H bonds in two different receptor-accessible states as compared with the SARS-CoV (51, 52). The uniqueness of the SARS-CoV-2 Spike protein is summarized in **Table 1**.

**Table 1:** Unique features of SARS-CoV-2 S protein and its implications in therapeutic purposes.

Features of SARS-CoV-2	Functions/Implications	References
The SARS-CoV-2 S protein has a unique RRAR sequence absent in SARS-CoV or other Beta CoVs lineage	This feature of the S protein is speculated to enhance the infectivity of the virus in human population	(24, 56)
The SARS-CoV-2 S protein has a polybasic insert which is absent in the β-CoVs. This insert is enclosed in ∼20 amino acids that resembles the toxin staphylococcal enterotoxins B (SEB)	Increases the binding affinity, probably causes the cytokine storm, and Multisystem Inflammatory Syndrome in Children (MIS-C)	(51)
The SARS-CoV-2 and SARS-CoV exhibits difference in residues that helps in formation of salt bridge between the Lys417 and Asp30 of SARS-CoV-2 S protein and ACE2, respectively	The stable binding enhances the viral entry into the host. Disruption of this interface could be a potential drug target.	(57)
SARS-CoV-2 S protein has a unique N- and O-linked glycosylation which is absent in the SARS-CoV.	Helps in the camouflage of COVID-19 from host defense systems.	(53)
Peptide markers unique to SARS-CoV-2 were identified from Spike (markers 6, 11, 13, and 21) and Nucleocapsid (markers 3 and 6).	The markers can be used as a complementary assay alongside with RT-qPCR	(58)
The SARS-CoV-2 has more number of atomic interactions with the hACE2 as compared to the SARS-RBD	The difference in interactions can shed light on the development of therapeutics against SARS-CoV-2	(59)
The S protein of SARS-CoV-2 has a higher number of helices and sheets (4 helices and 10 sheets) compared to the SARS-CoV (2 helices and 5 sheets).	The hotspot amino acid residues can be targeted to block the interaction with ACE2.	(52, 55)

## SARS-Cov-2 Spike and Host Protein Interactions

The entry of SARS-CoV-2 is mediated mainly through the viral S glycoprotein, S1 in particular, by binding to the host cell surface receptor *h*ACE2 (31, 60). Cleavage of the S1/S2 and S2 subunits, also known as priming, by the host proteases, is a crucial factor that determines the pathogenicity, structural flexibility, and tropism (23, 61). The cleavage site at the S1/S2 boundary also harbors a polybasic furin cleavage site, which is a hallmark of highly pathogenic viruses, such as avian influenza (62). The interaction of SARS-CoV-2-S1 with *h*ACE2 has a low dissociation constant (Kd), indicating a stronger interaction with *h*ACE2 compared with SARS-CoV-S (22). Interestingly, SARS-CoV-2 infection in the intestine with life-threatening cases is comparatively lower than that of lungs although the *h*ACE2 is vastly over-expressed in small intestine enterocytes compared with lung. This could be explained by the interaction of the highly abundant human defensin 5 (HD5) (a 32-residue amphiphilic antimicrobial peptide secreted by Paneth cells) with *h*ACE2, which have a fairly strong affinity (39.3 nM) that blocks the binding of S1 protein to *h*ACE2 (63).

The crystal structure of the SARS-CoV-2 Spike and hACE2 complex shows that a ∼24 amino acid helix of *h*ACE2 is associated with the RBD-*h*ACE2 interaction (22, 55, 64). The precise structural basis of RBD-*h*ACE2 interactions could provide valuable information to develop Spike-based vaccines or Spike targeting drugs (65). For instance, a 23-mer synthetic amino acid (SBP1) derived from the *h*ACE2 α1 helix was shown to have a Kd of 47 nM comparable to *h*ACE2 and SARS-CoV-2-RBD binding with Kd 14.7 nM (6, 66), indicating that SBP1 could inhibit the entry of the virus into the host cell if high concentration of SBP1 is used to outcompete the S protein. Because SBP1 is endogenous and derived from humans, it would be recognized as endogenous by the immune system. We have also designed peptides that may potentially block the RBD-*h*ACE2 (67); however, the new mutations in the RBD domain is a challenge that may affect the efficacy of these designed peptides.

Apart from *h*ACE2, other human proteins can also act as receptors for SARS-CoV-2 RBD-mediated attachment and entry into human cells. It has been recently reported that entry of SARS CoV-2 into the host cells is also mediated through the transmembrane receptor Neuropilin-1 (NRP1) (68). Because NRP1 is expressed largely in the CNS, it has been proposed as a route for SARS-CoV-2 into the host brain, which might explain the neurologic manifestations of COVID-19 (69). Other receptors involved in the entry of SARS-CoV-2 are olfactory receptor OR51E2 (70) and heparan sulfate (HS) (71). In addition, tumor marker CD147 (basigin) had been initially reported as a Spike receptor (59), which was later found not to have a direct interaction with SARS-CoV-2 Spike (59). Another receptor, AGTR2 (angiotensin II receptor type 2) that is highly expressed in the lung, has also been proposed to be a key receptor in the lungs for the entry of SARS-CoV-2 with higher binding affinity than *h*ACE2 (72). The Spike protein also interacts with GOLGA7 and ZDHHC5 acyl-transferase complex that promotes cytosolic tail palmitoylation, which could be a potential drug target (73).

## New Strains and Spike Mutations

Although there are several variants emerging from the original Wuhan strain, the following three lineages have been identified to be more infectious: lineage B.1.1.7 (WHO label: Alpha variant) identified in the UK, the B.1.351 (WHO label: Beta variant) in South Africa, and the P.1 (WHO label: Alpha variant) from Brazil (3). The three lineages have unique and common mutations on the RBD that interacts with the *h*ACE2. The N501Y mutation emerged in all three lineages. The B.1.1.7 shows key mutations N501Y, P681H, and H69-V70del. For B.1.351 and the P.1 lineages, the key mutations are N501Y and K417T, and E484K. These gain-of-function mutations increase the binding interaction of Spike RBD with *h*ACE2 and also escape from important antibody classes (3, 74). These immune escape variants could also cause reinfections and reduce vaccine efficacy (75). The increased affinity of the S mutant protein with *h*ACE2 was also identified in the cluster-five variant, initially identified in the farmed minks in Denmark, which have an Y453F residue mutation in the RBD. These mutations increased the binding to *h*ACE2 ~4 folds higher than the Wuhan strain but did not decrease the immune response to previously infected individuals, indicating that the variants strived for increased transmissibility rather than immune escape (76).

Until April 2021, several mutations in the Spike protein have been reported from different countries as described in **Table 2**. These adaptive mutations are making the virus more virulent with higher transmission rate, posing a question on the efficacy of the currently available Spike protein-based vaccines (78, 79). Besides, the precise mechanism of vaccine-induced thrombotic thrombocytopenia found in Spike-based ChAdOx1 nCoV-19 (AZD1222)/Oxford–AstraZeneca (80, 81) and Janssen COVID-19/Ad.26.COV2.S (82) COVID-19 vaccines are yet to be identified.

**Table 2:** Important SARS-CoV-2 strains and their adaptive mutations in the Spike protein.

Country of detection	Lineage/Strain	Classification	1^st^ Detected	No of Countries reported	Mutations in Spike protein	Transmission rate	References
China	**A 1-3 and B** (Wuhan-1/China)	NA	24-Dec-2019 to 5-Jan-2020	Worldwide	NA	-Moderate to higher transmission rate and disease severity	(28, 77)
United Kingdom	**B.1.1.7** (501Y.V1 variant, 20I/501Y.V1, UK COVID variant) (WHO label: **Alpha** variant)	VOC	20-Sep-20	129	**7 mutations:** N501Y, A570D, D614G, P681H, T716I, S982A, D1118H	-Higher transmission rate and disease severity -Moderate neutralization efficacy of convalescent sera or vaccines	(78, 79)
					**2 deletions:** H69-V70del, Y144del		
					**Key mutations:** N501Y, P681H, H69-V70del		
South Africa	**B.1.351** (501.V2 variant, 20C/501Y.V2, South African COVID-19 variant) (WHO label: **Beta** variant)	VOC	11-May-20	88	**9 mutations:** L18F, D80A, D215G, R246I, K417N, E484K, N501Y, D614G, A701V	-Higher transmission and reinfection rates -Significant neutralization efficacy of convalescent sera or vaccines	(78, 79)
					**1 deletion:** LAL 242-244 del		
					**Key mutations:** N501Y and K417N, E484K (escape mutation)		
Brazil	**B.1.1.248 or P.1** (B.1.1.28.1, 20J/501Y.V3 variant, K417T/E484K/N501Y) (WHO label: **Gamma** variant)	VOC	03-Nov-20	50	**12 mutations:** L18F, T20N, P26S, D138Y, R190S, K417T, E484K, N501Y, D614G, H655Y, T1027I, V1176F	-Very high transmission and reinfection rates -Significant neutralization efficacy of convalescent sera or vaccines	(78, 79)
					**Key mutations:** N501Y and K417T, E484K (escape mutation)		
India	**B.1.617**	VOI	25-Feb-21	1	**3 mutations:** L452R, E484Q, D614G	NA	NA
					**2 deletions:** del681, del1072		
India	**B.1.617.1** (WHO label: **Kappa** variant)	VOI	01-Dec-20	39	**5 mutations:** L452R, E484Q, D614G, P681R, Q1071H **Key mutation:** E484Q	NA	NA
India	**B.1.617.2** (WHO label: **Delta** variant)	VOC	7-Sep-20	65	**6 mutations:** T19R, L452R, T478K, D614G, P681R, D950N	NA	NA
					**1 deletions:** del157/158		
					Key mutation: T478K		
India	**B.1.617.3**	VOI	13-Feb-21	4	**5 mutations:** T19R, L452R, E484Q, D614G, P681R	NA	NA
					**Key mutation:** E484Q		

The table is prepared based on literature and information available at https://outbreak.info/situation-reports (accessed on 18-5-2021) and https://covdb.stanford.edu/page/mutation-viewer/#variants.with.adaptive.mutations (accessed on 18-5-2021).

VOI, variant of interest; VOC, variant of concern; NA, not available.

## SARS-Cov-2 Spike in Diagnostic Development

Among the main structural proteins of SARS-CoV-2, Spike (S) and nucleocapsid (N) are highly immunogenic. While the S protein is involved in the attachment of the viral particle to the host cells, N protein is involved in the viral RNA replication, packaging, and transcription (83). The gold standard method for detection of SARS-CoV-2 is whole-genome sequencing (WGS) from viral blood culture (3, 84). However, application of WGS is limited because of its cost and turnaround time. Therefore, amplification of the viral nucleic acid through reverse transcription polymerase chain reaction (RT-PCR) is the current standard for diagnosis of SARS-CoV-2, using ORF1ab and N gene of SARS-CoV-2 (84). Primer selection and RT-PCR reaction optimization are of key importance to avoid false-negatives, false-positives, and primer dimer formation (85). A recent study has shown that two primer sets (CDC_N2 and CDC_N3) from CDC produce false positives (Ct<37) even in the absence of the cDNA template. In another report, in-house designed SARS-CoV-2 RNA-dependent RNA polymerase (RdRP), N, E, and Sgenes specific primers to amplify 100 to 120 bp amplicons show no false-positive results (86).

Detection methods that rely on non-invasive sampling are preferred for large-scale screening. Amplification of the SARS-CoV-2 Spike gene from saliva samples has shown promising results (87); therefore, saliva is a good choice for sampling because salivary glands, gingiva, oral mucosa, as well as the tongue, could serve as hosts for SARS-CoV-2 due to the expression of *h*ACE2 receptor (88, 89). Furthermore, a recent study also suggests that RT-PCR diagnosis for SARS-CoV-2 using saliva samples is more sensitive than the nasopharyngeal or nasal swabs (90). However, there could be increased false-negative results due to the novel SARS-CoV-2 variants for which the primers are not designed properly. Mutation in the Spike gene, such as 69‐70del that is amplified by RT-PCR has shown to affect specificity and sensitivity of the assay (91). Similar results may also be observed for other genetic variation in the RBD sites if the 132 publicly available primers and probes are used to amplify the Spike gene of SARS-CoV-2 (92). Mass screening and sero-surveillance require rapid testing kits rather than RT-PCR (93). Spike protein can be used for such screening. Point of care lateral flow immunoassay (LFIA) can detect IgM and IgG antibodies against SARS-CoV-2 S protein within 15 minutes from blood samples with 88.66% sensitivity and 90.63% specificity (93). IgM, the first line of defense, against a viral infection is generated earlier than the long-term adaptive IgG that serves as immunological memory (94). IgM antibody against SARS-CoV-2 infection can be detected between 5 and 10 days, while IgG is detected between 14-21 days (95). Because SARS-CoV-2 and SARS belong to the same family, IgM detection could indicate recent exposure, whereas IgG could indicate otherwise (96). Hence, IgM- and IgG-based detection could provide brief information on the infection timeline. ELISA-based diagnosis for detection of IgM and IgG antibodies against nucleocapsid protein and S protein from confirmed that COVID-19 patients have shown that Spike protein-based ELISA has a significantly higher rate of positive results (97). In another approach, graphene-based field-effect transistor (FET) biosensing-coated antibodies against SARS-CoV-2 S protein exhibited high sensitivity (1 fg/ml) to nasopharyngeal swab (98). FET based biosensing has several advantages, such as instantaneous measurements at low concentrations, which make it ultrasensitive and a promising candidate for onsite detections.

Recent advances in the time resolved fluorescence (TRF) ELISA using monoclonal antibodies against different S1 subunit epitopes showed high specificity (99%) but with lower sensitivity (66%) when nasopharyngeal swab samples are used (99). Rapid detection methods for SARS-CoV-2 S1 subunit have also been developed using *h*ACE2 that forms a highly specific matched antibody pair with S1-mAb in a lateral flow immunoassay (LFIA) (100). The pros of this method include the non-cross reactivity with MERS or SARS CoV S1 subunit. Label-free electrochemical sensor that works on interruption of redox conversion in the presence of SARS-CoV-2 antibodies is also developed. It is a paper-based sensor with immobilized S RBD on the hydrophilic ePAD that selectively binds to the IgG and IgM produced against SARS-CoV-2 (101).

## SARS-Cov-2 Spike as a Therapeutic Target

**Figure 5:**
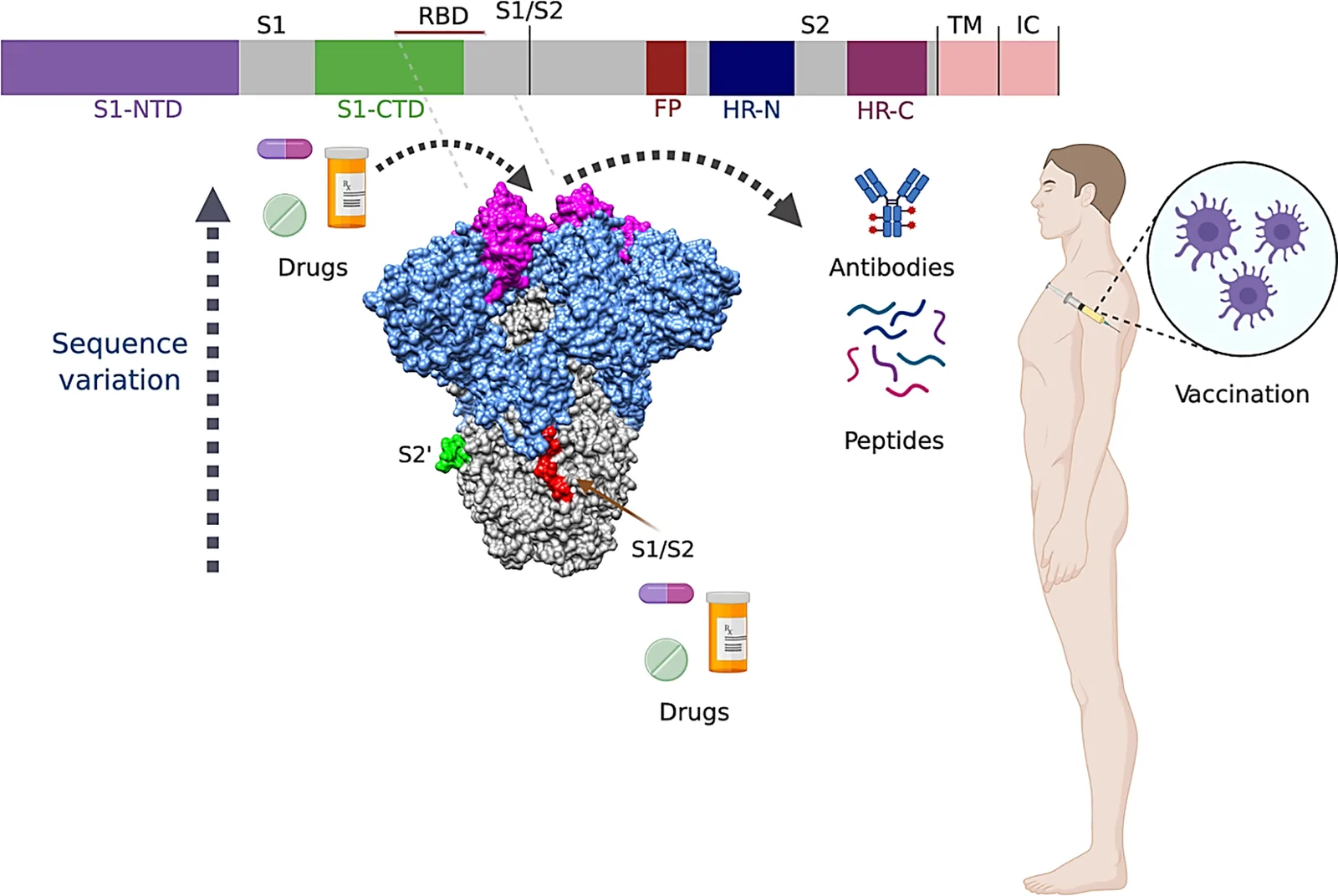
Summary of Spike protein targeted therapeutics. Sequence variability increases in the sense of S2 (gray) and S1 (blue) domains, and reaches its apices on the RBD (dark pink). Regions used for drugs, peptides, antibodies, and vaccine development are indicated with dark gray arrows.

The structural, functional, and antigenic characteristics of the S protein make it a good candidate for development of vaccines (11, 28, 31, 35), antibodies (102–106), and drugs (107–109) against SARS-CoV-2 (**Figure 5**).

Although sequence conservation of S protein makes it a good candidate for broad spectrum vaccine development against various CoVs, its mutations among various CoV strains pose a challenge. In many cases, Spike RBD of different CoVs, including MERS and SARS-CoV strains, shows a great antigenic capacity and induces neutralizing activity in infected animals and *in vitro* experiments (30, 40, 110–113). The conserved subsequence “KRSFIEDLLFNKV” found in many CoVs may be used as a peptide vaccine candidate. However, because the Spike RBD is a hotspot for mutation, there is always a risk of developing escape mutations in such conserved sequences (40). Another strategy for SARS-CoV vaccine development could be the use of full-length S protein to induce T-cells neutralizing antibodies (110, 114, 115), but, on the other hand, it has been reported that it may cause harmful immune responses causing liver injuries (110, 116).

The development of specific monoclonal antibodies (mAbs) against specific CoV strains is another way of controlling CoV infections (117). The use of Spike RBD region has been described for this purpose by several authors, where a single intrasplenic injection of plasmid DNA encoding the Spike region of interest is used for generating the desired immunity response (31, 118). For SARS-CoV-2, novel immunoglobulin (corresponding to RBD) against the SARS-CoV-2 could be screened from the peripheral blood of recovered patients using enzyme-linked immunosorbent assay (ELISA) and molecular cloning techniques to produce the desired mAbs for therapeutic purpose (106, 119). Moreover, the IGHV3-53 antibodies that is generated during SARS-CoV-2 infection targets RBD through alternated binding site and may be engineered for more effective neutralizing therapeutic antibody against SARS-CoV-2 infection (120). Drug development that involves S protein is mostly related to blocking its interaction with cleavage proteases and host cell surface receptors. Several *in vitro*, *in vivo*, and *in silico* (67, 121) studies with drug-like peptides (122) focusing on the RBD interaction with *h*ACE2 (108) and other surface proteases (123–125), surface host-cell receptors, and furin S1/S2 cleavage site have been reported with promising potentials (124, 125). However, except for a few cases, most of these studies that aim to block the interactions of RBD and S1/S2 with their respective host-cell receptor or protease using small molecules are mainly *in silico* (53, 126). In **Table 3**, we have summarized the important *in vitro* studies from the Coronavirus Antiviral Research Database (CoV-RDB) targeting S protein of SARS-CoV-2 for development of therapeutics.

**Table 3:** Promising therapeutics targeting SARS-CoV-2 Spike protein.

Preclinical Studies						
Virus	Type	Compound	Target	Study type	IC50 (µM)	Reference
**SARS-CoV-2**	Antibody	MAb-SARS-CoV-2-311mab-31B5	S-ACE2 interaction	Biochemistry	0.0002	(106)
**SARS-CoV-2**	Antibody	MAb-SARS-CoV-2-311mab-32D4	S-ACE2 interaction	Biochemistry	0.0003	
**SARS-CoV-2**	Peptide	hrsACE2	S-ACE2 interaction	Blood vessel organoid	<0.1	(106)
**SARS-CoV-2**	Peptide	hrsACE2	S-ACE2 interaction	Kidney organoid	>0.1	
**SARS-CoV-2**	Peptide	hrsACE2	S-ACE2 interaction	Vero E6	<<0.1	
**SARS-CoV-2**	Peptide	hrsACE2	S-ACE2 interaction	Vero E6	<<0.1	
**SARS-CoV-2**	Small molecule	Chloroquine	Entry (S protein)	Entry assay	6.8	(106)
**SARS-CoV-2**	Small molecule	Imatinib	Entry (S protein)	Entry assay	4.9	(127)
**SARS-CoV-2**	Small molecule	Chloroquine	Entry (S protein)	Entry assay	3.9	
**SARS-CoV-2**	Small molecule	Chloroquine	Entry (S protein)	Entry assay	12	(128)
**SARS-CoV-2**	Small molecule	Chloroquine	Entry (S protein)	Entry assay	9.3	

## SARS-Cov-2 Spike and Vaccine

Most of the vaccines currently under development, clinical trials, or in use, aim to prevent the uptake of viral particles *via h*ACE2 receptor and also induction of neutralizing antibodies (nAbs) (129). Therefore, use of SARS-Cov-2 S protein in vaccine development could serve two purposes: inhibition of receptor binding as well as viral genome uncoating (130). This is because the Spike subunit is comprised of S1 and S2 with distinct functions, where S1 mediates the *h*ACE2 receptor binding and S2 mediates the fusion and uncoating of the viral genome into the host cells (110). Furthermore, S protein with or without the presence of other structural proteins is the major inducer of nAbs and T-cell responses (110, 131). Thus, S protein is one of the most promising candidates for development of vaccines against SARS-CoV-2 (130). Additionally, self-assembling protein nanoparticles that block the Spike RBD and stabilize the Spike are also reported to be a potential vaccine candidate (116).

The broad-spectrum vaccine against SARS-CoV and MERS-CoV has been proposed based on the similarities in T-cell epitopes that could provide cross-reactivity (132). However, the S1 subunits of S protein of SARS-CoV-2 and SARS-CoV are variable, suggesting that the vaccines against SARS-CoV would be probably ineffective against SARS-CoV-2. Nevertheless, considering the high genetic similarity among the SARS-CoV and SARS-CoV-2, broad-spectrum vaccines against coronavirus infections could be explored (30, 130). The development of a universal vaccine against the coronavirus is essential to avoid future outbreaks from novel coronavirus species and strains (133). Variegated T-cell in subjects unexposed to the SARS-CoV-2 was found to be reactive to SARS-CoV-2 peptides (134–136). Such cross reactivity could be due to previous exposures to Human CoVs (*h*CoVs) that cause common cold with mild respiratory symptoms (136). The cross-reactivity could be based on the hCoV groups. hCoVs are grouped into group I (eg, hCoV-229E) and group II (eg, hKU1, MERS-Cov, SARS-CoV-1 and -2) (137). Immune responses to a recent infection by any of the group II CoVs could provide cross-protection or milder/asymptomatic infection upon subsequent exposure to the other CoVs within the group II (137). Between SARS-CoV-2 and SARS-CoV, there are similarities in phylogeny, genome sequence (~80%), binding to hACE2 as receptor for entry, and also exhibit false-positives in serology assay (137–139). In contrast, other studies have found weak cross-neutralizing despite the common cross-reactive response (138). Moreover, it is reported that only a minor fraction of the epitopes recognized in cross-reactive responses might be neutralizing epitopes (138). Currently, there are a variety of vaccines that have been approved and used in different countries that use the Spike protein as well as mRNA technology (**Table 4**). Additionally, with the emergence of novel SARS-CoV-2 variants, vaccination studies are still under investigation for a broad-spectrum coronavirus vaccine with high efficacy (78, 150).

**Table 4:** Currently approved Spike protein (S) -based important COVID-19 vaccines and their efficacy on Spike mutant strains.

Vaccine and country	Pharmaceutical formulation	General Efficacy	Efficacy on mutant strains				Reference
			Disease prevention D614G and B.1.1.7	Infection preventionD614G and B.1.1.7	Disease prevention B.1.351, P.1 and B.1.617	Infection prevention B.1.351, P.1 and B.1.617	
BNT162b2/Pfizer- BioNTech (USA) Approved	Lipid nanoparticle based full-length S protein mRNA vaccine	95%	91%	86%	76%	72%	(140, 141)
Ad26.COV2.S/Johnson & Johnson (USA) Approved	Adv based full-length S protein	>95%	72%	72%	64%	57%	(142, 143)
mRNA-1273/Moderna (USA) Approved	Lipid nanoparticle based full-length S protein mRNA vaccine	94.1%	94%	85%	79%	75%	(140, 144)
AZD1222/Oxford-AstraZeneca (UK) Approved	Adv based full-length S protein	>90%	75%	52%	10%	9%	(145)
Sputnik V (Russia) Approved	Adv based full-length S protein	91.6%	92%	80%	70%	61%	(146, 147)
ZyCoV-D/Zydus Cadila (India) Phase III	Plasmid DNA encoding full-length S protein and IgE signal peptide	–	–	–	–	–	(148, 149)

The efficacy of the vaccines on the mutant strains are taken from available data and modeled estimates of Institute for Health Metrics and Evaluation (http://www.healthdata.org/node/8584, accessed on 20-5-2021).

## Conclusion

The world is facing the third wave of SARS-CoV-2 infection. Spike is the key protein for the infection and transmission of this virus and the dynamic adaptive mutations in the Spike protein are making the virus more aggressive. Although rapidly developed Spike-based vaccines and therapeutics have been successful to some extent, the prevention and treatment of the infection are still major challenges. Deeper insights into the SARS-CoV-2 genome and proteome, especially its Spike protein structure, its uniqueness, function, and interactions with the host cell and immune system is essential to come up with innovative approaches to win the war against SARS-CoV-2 through the development of new vaccines and therapeutics. More importantly, one needs to better understand how and why the virus is evolving so rapidly and acquiring more aggressive characteristics. In this article, we have focused only on the Spike of SARS-CoV-2; however, other proteins of this virus should also be thoroughly investigated to understand these basic questions and to develop proper strategies to tackle this pandemic.

## Data Availability Statement

The original contributions presented in the study are included in the article/**Supplementary Material**. Further inquiries can be directed to the corresponding author.

## Author Contribution

Conceptualization, methodology, and design of the article: DB. Draft preparation: MI, RK, AC, BA, FA, ST, DR, KA, and DB. Technical inputs: VA, AG-N, MW, and PG. Editing and finalizing the paper: PG and DB. All authors contributed to the article and approved the submitted version.

## Conflict of Interest

The authors declare that the research was conducted in the absence of any commercial or financial relationships that could be construed as a potential conflict of interest
