# Supplementary material for: The Spike of SARS-CoV-2: Uniqueness and Applications
Source: Front Immunol. 2021 Jul 8;12:663912. doi: 10.3389/fimmu.2021.663912 (PMC8297464; doi:10.3389/fimmu.2021.663912)
Supplement: Supplementary file 1 [file DataSheet_1.docx]

**Supplement Table S1**. The number of articles available for each keyword search according to the given duration.

| **Keywords** | **Year of the first publication** | **Number of publications before 2020** | **Average of monthly publications before 2020** | **Number of publications after 2020** | **Average of monthly publications after 2020** | **Total of publications** |
| --- | --- | --- | --- | --- | --- | --- |
| Chloroquine | 1987 | 20 | 0.050505051 | 859 | 53.6875 | 879 |
| Diagnostic | 1976 | 977 | 1.850378788 | 6502 | 406.375 | 7479 |
| nCoV | 2013 | 10 | 0.119047619 | 1650 | 103.125 | 1660 |
| Spike | 1976 | 2061 | 3.903409091 | 3145 | 196.5625 | 5206 |
| Treatment | 1974 | 1169 | 1.297101449 | 14683 | 580.25 | 15852 |
| Vaccine | 1958 | 1338 | 1.798387097 | 3943 | 246.4375 | 5281 |

**Supplement Table S2.**  The number of articles available for Spike-specific keyword combination search according to the given duration.

| **Keywords** | **Year of the first publication** | **Number of publications before 2020** | **Average of monthly publications before 2020** | **Number of publications after 2020** | **Average of monthly publications after 2020** | **Total of publications** |
| --- | --- | --- | --- | --- | --- | --- |
| Antibodies and spike | 1984 | 694 | 1.606481481 | 1026 | 64.125 | 1720 |
| Diagnostic and spike | 1995 | 122 | 0.406666667 | 221 | 13.8125 | 343 |
| Docking and spike | 2003 | 17 | 0.083333333 | 311 | 19.4375 | 328 |
| Drug and targets and spike | 2003 | 21 | 0.102941176 | 185 | 11.5625 | 206 |
| Elisa and spike | 1993 | 149 | 0.459876543 | 157 | 9.8125 | 306 |
| Function and spike | 1986 | 102 | 0.25 | 172 | 10.75 | 274 |
| Genome and spike | 1987 | 354 | 0.893939394 | 439 | 27.4375 | 793 |
| Nucleocapsid and spike | 1983 | 275 | 0.619369369 | 362 | 22.625 | 637 |
| Pharmacological and spike | 2012 | 2 | 0.020833333 | 58 | 3.625 | 60 |
| Severe acute respiratory syndrome and spike | 2003 | 597 | 2.926470588 | 1376 | 86 | 1973 |
| Spike and origin | 1992 | 82 | 0.244047619 | 125 | 7.8125 | 207 |
| Spike and phylogeny | 1990 | 369 | 1.025 | 199 | 12.4375 | 568 |
| Spike and protein | 1976 | 1570 | 2.973484848 | 2702 | 168.875 | 4272 |
| Structure and spike | 1982 | 296 | 0.649122807 | 458 | 28.625 | 754 |
| Targets and spike | 1996 | 65 | 0.225694444 | 324 | 20.25 | 389 |
| Treatment and spike | 1979 | 154 | 0.31300813 | 622 | 38.875 | 776 |
| Vaccine and spike | 1988 | 508 | 1.322916667 | 890 | 55.625 | 1398 |
